# Malaysian primary care doctors' views on men's health: an unresolved jigsaw puzzle

**DOI:** 10.1186/1471-2296-12-29

**Published:** 2011-05-12

**Authors:** Seng Fah Tong, Wah Yun Low, Shaiful Bahari Ismail, Lyndal Trevena, Simon Willcock

**Affiliations:** 1 Department of Family Medicine, Universiti Kebangsaan Malaysia, Kuala Lumpur, Malaysia; 2 Discipline of General Practice, University of Sydney Medical Program, Sydney, Australia; 3 Medical Education and Research Development, Faculty of Medicine, University of Malaya, Kuala Lumpur, Malaysia; 4 Department of Family Medicine, Universiti Sains Malaysia, Kubang Kerian, Malaysia; 5 School of Public Health, University of Sydney, Sydney, Australia

**Keywords:** men's health, attitude of health personnel, family practice, health promotion, general practice

## Abstract

**Background:**

Men have been noted to utilise health care services less readily then women. Primary care settings provide an opportunity to engage men in health care activities because of close proximity to the target group (men in the community). Understanding attitudes towards men's health among Malaysian primary care doctors is important for the effective delivery of health services to men. We aimed to explore the opinions and attitudes of primary care doctors (PCDs) relating to men's health and help-seeking behaviour.

**Methods:**

A qualitative approach to explore the opinions of 52 PCDs was employed, using fourteen in-depth interviews and eight focus group discussions in public and private settings. Purposive sampling of PCDs was done to ensure maximum variation in the PCD sample. Interviews were recorded and transcribed verbatim for analysis. Open coding with thematic analysis was used to identify key issues raised in the interview.

**Results:**

The understanding of the concept of men's health among PCDs was fragmented. Although many PCDs were already managing health conditions relevant and common to men, they were not viewed by PCDs as "men's health". Less attention was paid to men's help-seeking behaviour and their gender roles as a potential determinant of the poor health status of men. There were opposing views about whether men's health should focus on men's *overall *health or a more focused approach to sexual health. There was also disagreement about whether special attention was warranted for men's health services. Some doctors would prioritise more common conditions such as hypertension, diabetes and hypercholesterolaemia.

**Conclusions:**

The concept of men's health was new to PCDs in Malaysia. There was wide variation in understanding and opposing attitudes towards men's health among primary care doctors. Creating awareness and having a systematic approach would facilitate PCDs in delivering health service to men.

## Background

The life-expectancy of men is shorter than women across both developed and developing countries by an average of 3.9 years [1,2]. The death rates for adult men across all regions in the world are higher than women [3,4]. Most causes of death in men such as cardiovascular disease, injuries, road traffic accidents, cancers, violence, war, infectious diseases (including HIV/AIDS) and chronic obstructive pulmonary diseases [3,4] are not male-specific and they are preventable or amenable to early intervention. A further analysis on six of these, accidents and their adverse effects, suicide, cancers, cardiovascular diseases, injuries, and chronic liver diseases among middle age adults in the major continents of the world has noted that men stand a higher risk of premature death than women [5]. In Malaysia, the difference in the average life expectancy between men and women is similar to the global pattern [6]. The life expectancy in Malaysia was about 71.7 years for men and 76.5 years for women in 2007 [6]. The common causes of death in Malaysian men are similar to the trends mentioned above, with cardiovascular disease and transport accidents being the top two [7]. Men in Malaysia also suffer high prevalence of many chronic disorders and health risk factors. In the 2006 national health morbidity survey, the high prevalent health morbidities include hypertension (33.2%), hypercholesterolaemia (18.6%), diabetes (12%), and smoking (46.4%) [8]. The prevalence of male-specific disorders was also high. Among other male-specific conditions, two-thirds of men aged 40 and above have moderate to severe erectile dysfunction, 19-29% have moderate to severe lower urinary tract symptoms [9].

Having noted the potential for early intervention in these male-predominant causes of death, early identification of the related health risks becomes important. These health risks include smoking, poor dietary intake, diabetes, hypercholesterolaemia and high risk behaviours such as addiction, violence and reckless driving. However, the topic of "men's health" is commonly associated with male -specific conditions such as urological and sexual disorders. The concept of men's health, which encompasses all unique factors affecting these health risks, is relatively new to many countries, including Malaysia [10], and might be less well understood by health care providers [11]. Unique factors that have been implicated in the health of men include masculine attitudes underpinning their help-seeking behavior [12-14], adverse social determinants surrounding men [15] and more importantly, the un-matched needs of men in service delivery [16]. The understanding of these unique factors and the concept of men's health is important in order to address men's health issues. This importance is even more so among primary care providers because they have significant contact with the community [17] and are potentially the main care providers to men. For example, tailoring care to the needs of men and understanding their help-seeking behaviour may result in better engagement of men in health services [18-20] such as cardiovascular risks assessment, life-style health checks, discussion of psycho-social issues, identification of sexual health concerns and many more [20,21].

In a survey of Asian physicians' attitudes to the concept of men's health in Singapore, Korea and Taiwan, Yates et al. concluded that the concept of men's health was less clear than women's health as viewed by physicians [11]. However, the concepts of men's health in the survey included only bio-medical aspects of men's health. Some have argued that 'men's health' should go beyond the biomedical aspects and also encompass psychosocial aspects [22-24]. Using such a questionnaire survey might also miss the depth and breadth of doctor's views about the concept of men's health and their help-seeking behaviour.

On the issue of health care providers' perception of men's help-seeking behaviour, early studies in 1998 and 2002 revealed that men were thought to be reluctant to accept and present to health care services unless necessary [25,26]. Men were noted to uphold their masculine image by being strong, stoic, able to take care of their own health and becoming the 'serious user of health service'. Most of these behaviours were seen as undesirable [25,26]. As men's health research progresses, the stereotyping of these men's help-seeking behaviours has been challenged and regarded as unhelpful in advancing men's health service [27,28]. Instead, men's decision in help-seeking is highly complex and greatly influenced by their social contexts, which are dynamic and changing over life stages [28]. More recent study exploring doctors' perception about men's help-seeking behaviour found that doctors still perceive poor help-seeking behaviour of men as the result of the need of men to uphold stereotypical masculine image [29,30] rather than as the result of their interactions with the societies.

In Malaysia, primary care outpatient services are mainly provided by government-funded public health clinics and fee-for-service private clinics. In public clinic settings, women and children's health has been a priority with sections devoted to these services in the premises of the clinics [31]. On the other hand, health services for men are included under the umbrella of general outpatient services, making it less likely that care will be tailored to the needs of men. Service delivery in the private sector is also unlikely to specifically adapt care to the needs of men [31]. In order to improve health care delivery to men in primary care settings, it is vital to explore the primary care doctors' perception of men's health and men's help-seeking behaviour besides from the lay person's perspective.

Therefore, this paper aims to explore primary care doctors' views of men's health and help-seeking in Malaysia using a qualitative approach. The findings presented are part of a larger project aimed to explore the determinants of decision making by primary care doctors in undertaking men's health screening in Malaysia.

## Methods

A qualitative method was used because it enables an in-depth understanding of primary care doctors' opinions and allows doctors to describe them freely without restriction. Furthermore, the concept of men's health is relatively new to many Malaysian primary care doctors and the identification of major components and constructs of this issue is fundamental in the first instance.

### Study setting and recruitment

In Malaysia, primary care outpatient services are delivered through government-funded clinics (public clinics) and private self-funded clinics (private practice). These two sectors vary enormously in the way they deliver care. Consequently, we selected primary care doctors purposively to represent the public and private; the urban and rural; the male and female; those with basic degrees and post-graduate training. The invitation to participate was done through different sources. Invitation letters were sent to the 200 private PCDs practicing around Kuala Lumpur and Petaling Jaya and from the list of contacts provided by Academy of Family Physicians of Malaysia. For public clinics, contacts were sought through the chair of the Selangor division of Family Medicine Specialists Association (FMSA). All 25 members of FMSA in the Selangor division were invited. The heads of two primary care centers, one public health clinic and one academic primary care center, were also contacted to help in inviting the doctors from their respective centers to participate. These areas were chosen because of logistic reasons for focus group discussion or in-depth interview. Finally, three personal invitations were made to the key opinion leaders in primary care organisations by the researchers (SF). From all the recruiting strategies above, 20 PCDs responded from the invitation letters, 10 out of 25 FMSA members, 19 PCDs from contacts via head of clinics and all three key opinion leaders accepted the invitation. As much as possible, if the participant could agree on a common venue and time to meet, focus group discussions (FGDs) were arranged. Otherwise, in-depth interviews (IDIs) were held according to preference of the participants. In-depth interviews were organised for all three key opinion leaders. They were interviewed individually because they might exert significant influence on the group dynamics if they were to be invited for focus group discussions. We did not segregate male and female doctors in FGDs because by knowing that gender is relational [13], the interaction and stimulating nature within focus groups from different gender may yield richer data about the topic of men's health [32]. A total of 14 IDIs and 8 FGDs were conducted involving 52 doctors. None of the doctors involved had any formal training in andrology or men's health.

### Data collection

**Figure 1:**
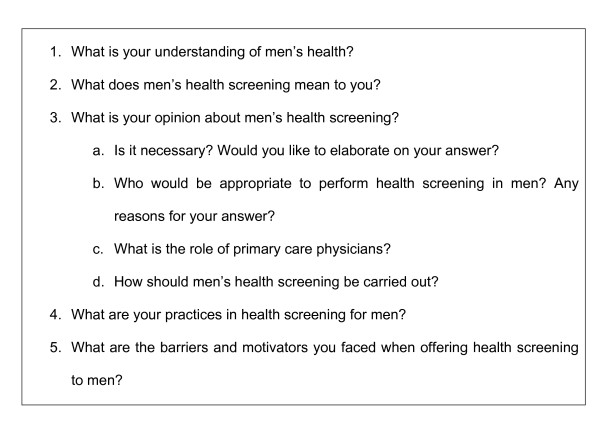
**Semi-structured interview guide used for both focus group discussions and in-depth interviews**.

FGD provides an opportunity for doctors to exchange ideas and stimulate further thoughts beyond their own original ideas, IDI allows expression of views that the participant may not want to reveal in the presence of their peers [33]. All FGDs and IDIs were moderated and conducted by SF. All sessions were conducted in English because English is the common language in the medical fraternity in Malaysia. Sessions were guided by semi-structured questions to stimulate discussion. (Figure 1) The questions were compiled to address the study objectives and were derived from the literature. All questions were open-ended, starting with "What is your understanding of men's health?" Free-flowing discussion was encouraged to gain unrestricted opinions on the topic of interest. Participants were briefed about ground rules to ensure confidentiality and the objectives of the study. It was emphasised that the discussion was not meant for assessment. The participants in the FGDs were kept to similar training backgrounds to avoid intimidating situations between the primary care doctors with post-graduate training and junior primary care doctors. Consent was sought for audio-recording before each IDI and FGD. A note taker assisted all FGDs to help with recording and taking note of who was conversing. All sessions were audio-taped and subsequently transcribed verbatim for analysis.

### Data analysis

The first three transcripts (2 IDIs and I FGD) were read repeatedly to gain an overall understanding of the interviews. This was followed by line-by-line coding of the transcript to identify in detail the concepts expressed by the participants. At the end of coding for each transcript, a memo was written to capture an overall impression of each interview in order to provide a distant view of what the participant's opinions are, as opposed to the detail analysis of line-by-line coding [34]. The transcripts and the summary of the concepts were read by WY and SW to ensure validity of coding. Concepts emerging from one transcript were compared with the subsequent transcript and sorted to identify the common themes. The themes were continuously refined by constantly comparing with further emerging concepts from subsequent analysis of transcripts. Data saturation was achieved after the analysis of the 11^th ^transcript (8 IDIs and 3 FGDs). Further analysis of the remaining 11 transcripts did not yield further new concepts. This method of constant comparison and sorting is a legitimate data analysis method in qualitative analysis [34]. Throughout the process of comparison, sorting and coding, memos were written to record and keep tract of emerging ideas about the concepts. It also acted as reflexive notes for researcher in order to minimise potential bias while interpreting the transcripts and keep the concepts grounded on the data [35]. The data management during the process of analysis was done with the assistance of QSR Nvivo8 software [36].

The study was approved by the Ethics and Medical Research Committee, Ministry of Health Malaysia (NMRR-08-1516-3079), the Medical Ethics Committee, University Malaya Medical Centre (679.28) and the Human Research Ethics Committee, The University of Sydney (03-2009/11490).

## Results

A total of 52 doctors from various backgrounds and experience participated (Table 1). The youngest practicing primary care doctor was 30 years old and was enrolled in a training program for the Master of Family Medicine, whereas the oldest was 69 years old and a practicing private primary care doctor. There were similar numbers of PCDs with basic degrees versus postgraduate training. A larger number of private doctors (8 private versus 2 public) were involved in IDIs because they were not able to accommodate their time for participation in focus group discussions.

**Table 1:** Demographic and practice characteristics of participants

Characteristics	Number of participants (n = 52)
Age range (years)	30 - 69
Male to female ratio	19: 33
Postgraduate to basic degree ratio	26: 49
Urban to rural practice ratio	41: 11

Five main themes emerged from analysis of their views about the practice of men's health in Malaysia. The concept of 'men's health' was new to many primary care doctors. There was much debate on the scope of men's health and doctors had opposing views about men's health.

### Fragmented views of men's health and men's health services are inadequately delivered

Men's health was often cited as a new term and concept. PCDs referred to the definition of health by the World Health Organization (WHO), which described health as complete physical, psychological, spiritual and social wellbeing [37]. Therefore, men's health would be the health issues of men in relation to those areas.

"You should follow the definition of WHO"

-41 year-old female public doctor

"...looking at the chapter itself, men's health is a new chapter to me"

-51 year-old male private doctor

PCDs described the provision of many services such as cardiovascular risk screening and treatment of chronic disease to male patients, but these services were done without awareness that it could be part of a men's health service. They were perceived as services rendered to any patient without being gender-specific.

"[U]ntil lately, our questions were just, you know...opportunistic screening and all that. We are not thinking about men's health"

-62 year-old male private doctor

This showed that there was no systematic approach to men's health care, particularly for preventive health activities such as smoking cessation, counseling, health screening and sexual health. Men's health was seen as a piece in an incomplete jigsaw puzzle. While not entirely foreign, it remained a challenge to primary care doctors to see it as a distinct entity. They knew something needed to be done but were not exactly sure what should be included.

"Most of the things [health care services provided], they know but they don't do it as part of men's health. They know is a part of medical consultation. There should be bits and pieces there inside a patient; you have all the pieces of **jigsaw puzzles **of men's health. The other problem is there are a lot of missing pieces around"

-62 year-old male private doctor

Many doctors also expressed concern about their skills in approaching men. They were competent in dealing with general issues, but were less confident about what to cover in men's health issues, more so if it was a sensitive area such as sexual health. They wished for better training in skills and knowledge.

"The minute they talk about...oh no!..they can't perform[sexually], you'll end up with a problem here in consultation room"

-58 year-old female private doctor

### Debating the scope of men's health: Holistic versus male-specific men's health

At one end of the spectrum of opinion, the PCDs thought that there should be a holistic approach to men's health. At the other end, some PCDs advocates seeing men's health as only related to male-specific disorders. Those taking a holistic perspective would include all aspects of health as defined by WHO. These would also include healthy relationships with family members and men's working environments. They also emphasized the need to look into men's quality of life. They noted that physical health, mental health and social health were interrelated while acknowledging the need to explore men's sexual health, because in the local society sexual health carries an image of manliness and masculinity. A man's image in the society could be jeopardized if he was known to have sexual dysfunction [38]. Therefore, sexual health has to be addressed alongside the other aspects of men's health, such as their psychological wellbeing and underlying medical problems.

"The patient might have diabetes, hypertension, and also the problem of erectile dysfunction which may affect his relationship, his social life and so on. It's all interrelated"

-38 year-old female public doctor

Being holistic about men's health also includes issues that are common to men or where men were at higher risk.

"My priority would usually be on the most prevalent disease that men faced, it should be cardiovascular [problems]. That is the most important thing that we need to look at."

-45 year-old male private doctor

This holistic scope of men's health was challenged by some participants. On the one hand, they agreed that care for male patients should include all aspects of their health as defined by WHO, but when referring to the term 'men's health', they would prefer referring it to male-specific conditions in order to differentiate men's health and general health. In their opinion, although cardiovascular health was a concern to men, it should still 'remain under' cardiovascular health, i.e. not to be included under men's health discussion.

"Partly...true also but I think now, when talk about men's health, because now cardiovascular diseases come under cardiovascular screening, so.... when we talk about men's health right now, it is more towards men's sexual organ disease like prostatism, prostatitis or sexual dysfunction"

-48 year-old male public doctor

Health issues were preferred to be demarcated into general health, women's health and men's health. Although the doctors preferred such division, they did recognise that sexual health, particularly erectile dysfunction, was associated with cardiovascular morbidities. They advocated holistic approaches to urological and sexual health and erectile dysfunction was seen as the indicator for men's overall health.

"So if this patient has ED, definitely this patient is prone for hypertension, diabetes. So, we are using ED as an indicator"

-47 year-old male private doctor

Although there was debate about the scope of men's health, the emphasis was still on men's sexual health. At one end, some PCDs would want to assess men from various aspects of health simultaneously including sexual health, whereas the others advocated that sexual health should be the focus of men's health with a holistic approach.

### Opposing views on the approach to men's health: Universal approach vs. special attention to men's needs

The proponents of a universal approach to men's health argued that men should be approached the same way regardless of their gender in non male specific conditions. In their consultations, men were considered as having similar health needs as women as far as common health issues are concerned. Some even questioned the reasons for having a separate entity conceptualised as men's health.

"Well, I think as the health care provider, services that we provide, we should be universal for male or female in whatever age groups, it should be universal"

-47 year-old female public doctor

Areas that require a different approach would be sexually related health matters.

"I agree with Dr. X, for general health, there should be no different but, if you talking about men's health [in relation to sexual health] then you should have a different approach"

-47 year-old male private doctor

"Most men are, at least I think, quite concern with the sexual health concern that relates to the masculinity, manliness, so that is another factor that we got to know about."

--62 year-old male private doctor

The opponents of a universal approach stated that men exhibited different help-seeking behaviour and hence needed special attention. Men would not present readily for health assessment or for trivial illnesses due to the prescribed image of masculinity in the society. Men also have different social priorities and responsibilities in life. For example, men would not attend health services during working hours because they placed a higher priority on work. Therefore, health service delivery to men has to take these factors into consideration.

"...this group of people [men] who need to be screened but do not seem to come for screening due to their responsibility...because men seem to think perhaps health screening is not one of the priorities. The priority is to earn money and be the breadwinner for the family. That's more priority to them. So perhaps you should have advocacy for men health, it will allow them to come and keep the opportunity for them"

-47 year-old female public doctor

### Opposing views on men's health service: Unnecessary emphasis vs. noting the importance

Some PCDs felt that specialised attention to 'men's health' should not be a priority. They felt that other existing specialties like occupational health, sexually transmitted disease, anti-aging medicine and cardiovascular health were already addressing issues related to men. Hence, it was not practical to have a category termed men's health.

"Aging medicine is already there, I don't have to talk about it. Whole lot of people addressing the issue here. These [men's health] are meaningless. If you are not practical, you forget it. There is no big deal about men's health."

-68, male, private clinic

There were more urgent health issues such as hypertension, diabetes and acute illnesses, which deserve greater priority. Specific men's health concerns such as sexual health were considered secondary to these common illnesses.

"...there are many other problems to settle...hypertension, diabetic rather than men's health, this one doesn't kill you..."

-48 year-old male public doctor

Men's sexual health problems were said to be uncommon, hence not cost-effective in terms of deserving emphasis.

"Men's health...I would think that it would be rather...not economical. The yield would be rather low. If... if you are talking about prostate cancer...the yield will not be there. Once in a while they (men) will come for erectile dysfunction but that would be pretty rare."

-38 year-old male private doctor

On the other hand, the contrary argument was made that men's health issues were intertwined and unique. Care should not be fragmented. These participants noted the effects and relationships between sexual health and common medical illnesses, social relationships and family systems. Men have important roles as the financial providers and companions to the family.

"... patients with ED problem also have the risk of having MI or having heart problem. So I think it's important"

-30 year-old female public doctor

"I not sure why but, maybe that's why we try to capture this group of people [men] who need to be screened but do not seem to come for screening due to their responsibility and other things that concern them out there."

-48 year-old female public doctor

Participants noted the special communication skills needed in approaching men and the need to be sensitive to their masculine image and social responsibility.

"Tackling the male [patients] is not simple. It is not similar with tackling the female patients."

-41 year-old female public doctor

They often advocated specific approaches to men's health service delivery, with special attention given to opportunities to address men's health issues. At present the health care system, particularly the public health system, has special services for maternal and child health.

"Well, I think as a health care provider, services that we provide, we should be universal for men or female whatever age group, it's should be universal. However to advocate and to capture men's interest, we might have to use different approaches for them [men] to use the services. Men seem to think perhaps health screening is not one of the priorities. So perhaps you should have advocacy for men health, it will, you know, allow them to come and know that, you know...we have an opportunity for them..."

-48 year-old female public doctor

### Men's help-seeking behaviour: an obstacle to men's health

In the IDIs and FGDs, negative remarks on men's help-seeking behaviour were often made. Negative help-seeking behaviour would include late presentation to health care and disengaging in preventive health activities. Often, the first impression was men did not readily present to health care providers for their health problems unless there was compelling reasons to do so.

Because it's [coming to see doctors] all voluntary. So usually the already sick will come to clinic rather than where the needs are.

-39 year-old female public doctor

This idea of "seeing a doctor is when you're sick"... maybe, it's true here because overall, you will see men will come [present to health care], you know, with a heart attack!

-44 year-old female private doctor

Men were noted to take an illness orientated approach and evaded the discussion of health checks. This had resulted in some doctors becoming cautious when initiating the discussion of health checks in order to avoid 'losing' their patients.

One may think "Wah, this doctor is really good, I come for one issue but he looks into me as a whole" But most of the patients, 9 out of 10 will be thinking, I come for a knee problem, my leg problem and he's looking at my third leg. You know what I mean? So it will sound as if is this doctor correct? I may be a subject of discussion amongst a group of patients, laughing about what kind of mentality that doctor has. I got to be very very careful because this is the Asian set-up

-50 year-old male private doctor

The doctors had the impression that men were ignorant to their health. This was attributed to the desire to be strong, stoic and having high threshold for tolerating symptoms of illness.

If you want to do screening for them. Nobody will come to your clinic. "What for? I'm healthy", They think they are strong.

-47 year-old male private doctor

They are not expressing their inner needs. They may be egoistic.

-41 year-old female public doctor

Only a few doctors shared the concern that these sets of behaviour were the results of the societal values and cultural norms. Being a man in the society, he is expected to be the breadwinner for the family. Men in the family are expected be strong and not to fall ill. Work and earn for the family became the priority rather than health. Time spent for health concerns was seemed to be unjustified especially when there was no symptom of sickness.

They think, "I'm the head of the family, I shouldn't fall sick"

-41 year-old female public doctor

They find time is very special for them because they're the breadwinner... That's why they do not come for screening voluntarily. It's time consuming [for the men].

-42 year-old female public doctor

The society viewed that only the sick men would see doctors. Men fear the consequences of knowing their illness and taking medication was a hassle for them.

The other thing is that this idea of seeing a doctor only when you're sick. It has long been ingrained in... all societies.

-44 year-old female private doctor

They don't want to see the consequence of the disease, they fear knowing that they have health problems.

-46 year-old female public doctor

Men also noted to be reluctant in accepting family members' advice in their help-seeking behaviour both for illness presentation and health checks. This reflected the hierarchy of power struggle of men in the society.

Advising your husband to come to the clinic? Oh my God! It's not that easy. Man is dominant in the family. They have the say in family. So, wife's opinion is secondary perhaps...

-36 year-old female public doctor

Men who portrayed positive help-seeking behaviour were minority. They were older, having family responsibility, perceiving a threat in their health and better informed on health issues.

If one of their family members or close friends has a serious disease, so they come forward and requesting for the doctors to do the necessary screening.

-42 year-old male private doctor

Men who have live longer do realize that they are the breadwinner of the family and they have to stay much longer to raise their children and everything. Then, they are aware that they have to look after their health.

-51 year-old male private doctor

Gender of the doctors is thought to have an influence on how they view men's health and their help-seeking behaviour. Contrary to the assumption, a review of our data did not yield substantive differences between male and female doctors. Both among male and female doctors themselves had opposing views of men's health. Both male and female doctors shared much similarity in how they viewed men's help-seeking behaviour.

## Discussion

This study showed a wide variation in opinions about men's health among Malaysian primary care doctors. The concept of men's health was relatively new to participants. The help-seeking behaviour of men was thought to be the obstacle to improving men's health. There was also debate on the scope, the approaches and the importance of men's health. The opposing opinions about men's health suggest that there was a varying degree of exposure to the concept of "men's health" among primary care doctors.

Many doctors were unaware of the formal definition of men's health. Hence, they drew their understanding from the definition of health as stated by the World Health Organization (WHO) [37] and see it from the men's perspective. Although the WHO concept of health is holistic, it does not take gender differences into consideration. These are in contrast with the definitions of men's health provided by various men's health organizations [22,23]. Almost all men's health definitions have emphasised the important to acknowledge the unique roles of men in society [13,22,23] and their social circumstances [15]. These social roles and social circumstances have significant influence on the male image of masculinity and contribute to poor help-seeking behaviour among men [39]. In our study, the doctors noted the need to address common health problems that are prevalent among men or for which men are at a higher risk, and poor help-seeking behaviour might be the reasons for such problems. Men were noted to be ignorant about their health, but they seldom discussed the underpinnings of poor help-seeking behaviour. They related poor help-seeking behaviours to the adverse effect of the men trying to maintain their masculine image. Only a few attributed men's help-seeking behaviours to social-cultural influences. Current men's health discourse recognises that adverse social environment [15], the need to maintain a masculine image in the society [12,14,40] and the expectations of society on men [39] are important issues underpinned poor men's help-seeking behaviour. Although men contribute to the construction of their help-seeking behaviour, the society also helps significantly to the maintenance and (re)construction of their 'negative' help-seeking behavior [39].

The doctors' perception of masculinity and its adverse influence on help-seeking behaviour may not reflect how men in the community perceived and experienced it. In a study interviewing men aged 40 in Malaysia, men were noted to value health highly as an important asset. Men did not ignore health but engaging in healthy life-styles and appropriate help-seeking for health related issues faced many challenges. Avoiding engagement in health care was noted as a way to demonstrate their superiority in the society and guard their manly image because the society expected them to be so. The important life priority for men was job and wealth. Engaging in health care only became the priority when men aged, have family and faced with illness experience [38]. These findings confirmed an early study about Malaysian men's opinion on the attributes of a 'real man'. A 'real man' in the society should possess 'a good job', 'be a family man', 'have lots of money', 'be seen as a men of honour' and 'be in control in life' [41]. These attributes have great similarity with the some of the classic features of hegemonic masculinity [27]. 'To be seen as a man of honour and 'to have a good job' were noted to be associated with poorer help-seeking behaviour. On the other hand, 'to be a family man' was associated with positive help-seeking behaviour [42]. Study looking at influence of South Asian's culture on men's help-seeking behaviour also noted that some attributes of masculinity, such as 'to be a responsible family man', act as motivators to appropriate help-seeking behavior [43]. Study by Sloan et al. also noted that some men in the society justifying their healthy life-styles to demonstrate autonomy (an attribute of masculinity) [44]. Hence, it is a myth that all masculinity features have negative influence on help-seeking behaviour. Some could be mobilised to motivate healthy life-styles and appropriate help-seeking behaviour.

One participant had coined the term 'bits and pieces of jigsaw puzzle' as the metaphor for the current understanding of men's health among PCDs. The doctors might have all the pieces of the jigsaw but not be able to put them in a single picture. The oversight of the underpinnings of poor help-seeking behaviour might represent missing pieces in the unresolved puzzle of men's health. Therefore, the awareness of seeing men in their gender roles is important (thus seeing the complete jigsaw) rather than viewing them just as *male *patients from a bio-medical perspective.

The doctors who argued from the stance of the WHO definition of health would adopt the holistic approach in men's health; while those who adhered to a male-specific perspective would adopt a sexual health approach. However, at both ends of the debate spectrum, doctors noted the connectedness of bio-psycho-social issues. It appears that the debate was really about what the prime focus of attention should be when doctors talk about men's health. The definition of men's health advocates looking at all health issues related to life-styles, occupation, home environment and socioeconomic status that have a specific impact on men or boys [20,24]. It might be a misunderstanding that the scope of men's health centers on sexual and urological health. The misunderstanding is understandable because erectile dysfunction and male urological disorders are male-specific. The differing opinions about the scope of men's health were also documented in a quantitative survey from Asian countries. It showed that different specialties might have different opinions about the disease conditions and specialist areas associated with men's health [11]. In the Asian survey, more than 80% of the endocrinologists and cardiologists interviewed mentioned that their own specialties were the main focus for the treatment of men's health conditions. Acknowledging the morbidity and mortality patterns of men, and current understanding of men's health behaviour, it is appropriate to adopt a holistic and gender-sensitive approach to men's health. Clinician need to recognise the mutual influence between men and his societies. If we can align our perspective to see men in their gender role and acknowledge the potential impact of society on men's help-seeking, our focus should shift to address not only the male-specific disorders, men's individual health and psychology of men's help-seeking but also the societies surrounding men.

The doctors who noted the men's unique roles and responsibilities in society acknowledged the importance of having a specific approach to men's health service delivery. They advocated health services deliveries tailored to the needs of men rather than the current system which ignored gender sensitivity. Although there is no clear evidence that a gender-sensitive approach to men's health service delivery is more effective than a general approach [45], such gender-sensitive approaches to men's health service delivery were regarded as necessary by many commentators and reviewers on men's health [16,19,24,46]. It seems logical to have a gender-sensitive approach because their help-seeking behaviours differed from those of women. On the other hand, doctors who adopted the views of a universal approach to service delivery for both men and women might not recognise that such needs require a different approach when dealing with men. They understood what health is but were unaware of the different health care needs for men.

Although men's health issues may be better understood from gender-relations approach [13] and hence gender of the doctors may influence their opinion on men's health issues and men's help-seeking behaviour, an analysis of our data did not reveal such differences between male and female doctors. A review of gender influence on doctor-patient communication suggests that gender has minor influence and communication styles are more important issues to consider [47]. The gender of doctors also play a minor role in the decision making process of offering men's health check-ups [48].

The literature on primary care doctors' views on men's health is scarce. Despite advances in the men's health movement in the United Kingdom and Australia [49], and across the globe [10], men's health discourse is still relatively new to Malaysian primary care doctors. Hence, a qualitative approach was deemed more appropriate to explore the breadth of opinions and to capture as many diverse views as possible. While the content of the interviews and focus group discussions could possibly have been influenced by the relationship between participants and researchers [26], we believe the data gathered has an acceptable level of credibility because great efforts were taken to create a safe environment to encourage honest expression of opinions and views from the participants.

## Conclusions

The concept of men's health was new to many Malaysian primary care doctors and hence resulted in confusion about the best approach to caring for their male patients. Some doctors appreciate the specific needs of men in health, but many were unable to link specific men's health needs and health problems faced by men. Creating awareness and using a systematic approach that takes into the account general as well as gender-specific issues could prove very helpful for primary care doctors in providing effective health care deliveries to men.

## Abbreviations

PCD: Primary Care Doctor; IDI: In-Depth Interview; FGD: Focus Group Discussion; WHO: World Health Organization; FMSA: Family Medicine Specialists Association; ED: Erectile Dysfunction

## Competing interests

The authors declare that they have no competing interests.

## Authors' contributions

TSF conceived the idea, coordinated and conducted the study, analysed the data, and drafted the manuscript. WYL helped in refining the idea, supervised the study design, participated in data analysis and help to draft the manuscript. SBI coordinated part of the study, contributed to data analysis and drafting of the manuscript. LT helped in the conceptualized the idea for the manuscript and the draft of the manuscript. SM helped in supervising the study design, participated in data analysis and the draft the manuscript. All authors read and approved the final manuscript.

## Pre-publication history

The pre-publication history for this paper can be accessed here:

http://www.biomedcentral.com/1471-2296/12/29/prepub
